# Decisions of persons, the pharmaceutical industry, and donors in disease contraction and recovery assuming virus mutation

**DOI:** 10.1186/s13561-021-00320-4

**Published:** 2021-07-23

**Authors:** Kjell Hausken, Mthuli Ncube

**Affiliations:** 1 Faculty of Science and Technology, University of Stavanger, 4036 Stavanger, Norway; 2 Said Business School, University of Oxford, Park End Street, OX1 1HP Oxford, United Kingdom

**Keywords:** Pharmaceutical industry, Health, Patients, Donors, Safe versus risky behavior, Disease contraction, Recovery, Death, Drug development, Virus mutation, Subsidies, Game theory

## Abstract

**Background:**

The article develops an eight-period game between N persons and a pharmaceutical company. The choices of a donor and Nature are parametric.

**Methods:**

Persons choose between safe and risky behavior, and whether or not to buy drugs. The pharmaceutical company chooses whether or not to develop drugs. The donor chooses parametrically whether to subsidize drug purchases and drug developments. Nature chooses disease contraction, recovery, death, and virus mutation. The game is solved with backward induction.

**Results:**

The conditions are specified for each of seven outcomes ranging from safe behavior to risky behavior and buying no or one or both drugs. The seven outcomes distribute themselves across three outcomes for the pharmaceutical company, which are to develop no drugs, develop one drug, and develop two drugs if the virus mutates. For these three outcomes the donor’s expected utility is specified.

**Conclusion:**

HIV/AIDS data is used to present a procedure for parameter estimation. The players’ strategic choices are exemplified. The article shows how strategic interaction between persons and a pharmaceutical company, with parametric choices of a donor and Nature, impact whether persons choose risky or safe behavior, whether a pharmaceutical company develops no drugs or one drug, or two drugs if a virus mutates, and the impact of subsidies by a donor.

## Background

### Contribution

This article assesses the strategic choices of persons to engage in risky behavior and whether or not to buy drugs, a pharmaceutical company choosing whether to develop expensive drugs to combat disease, and a donor choosing parametrically whether to fund drug development and drug purchases for poor people in the face of declining aid flows and growing patient incomes.

An eight-period game is developed. A disease such as a HIV/AIDS or Covid-19 virus attacks a person or not, given that the person chooses risky behavior. The pharmaceutical company responds by developing or not developing drug 1 which the person buys or not and the donor subsidizes to a certain degree, or does not subsidize. The person responds positively and the virus is contained or it mutates. If it mutates, a drug 2 is or is not developed. Upon consuming or not consuming drug 2, the person recovers or dies.

The person’s expected utility is life. The pharmaceutical company’s expected profit follows from drugs being bought. The pharmaceutical company benefits if the person buys and consumes drug 1 or indeed drug 2 perpetually, like anti-retrovirus, or one off when the person recovers completely. The pharmaceutical company incurs costs of investing in drug research and development. The person pays for the drug or it is donor-funded through aid flows. Infected persons may suffer consequences such as losing their jobs or otherwise experience decreased life quality. The potential patient can also invest in acquiring knowledge on how not to contract the disease.

The article’s objectives and research questions are to determine the players’ optimal strategies, and how these strategies and the model parameters impact which of the seven outcomes emerges. The methods in the article are to solve the game with backward induction starting with period 8. In period 8 Nature chooses recovery versus death probabilistically. In period 7 a person buys drug 2, sponsored by a donor, if the benefits outweigh the costs. In period 6 the pharmaceutical company develops drug 2 sponsored by a donor, if the benefits outweigh the costs. In period 5 the virus mutates or does not mutate. In period 4 a person buys drug 1, sponsored by a donor, if the benefits outweigh the costs. In period 3 the pharmaceutical company develops drug 1 sponsored by a donor, if the benefits outweigh the costs. In period 2 Nature chooses disease contraction probabilistically. In period 1 the person chooses risky or safe behavior.

The model brings together persons which may contract a disease and purchase drugs, a pharmaceutical company which may or may not develop drugs, a donor which may or may not subsidize, a virus which may or may not mutate, and Nature which impacts disease contraction, recovery, and death.

The model’s assumptions are to consider diseases fulfilling three requirements. First, we assume that whether a person contracts the disease depends on whether the person chooses risky or safe behavior, e.g. using a condom against HIV or wearing a mask or keeping distance against Covid-19. Hence diseases are excluded which do not depend on the person’s behavior, which are genetic or hereditary, which are caused by the environment, or which depend on economic and political factors and societal trends outside the person’s control. Second, we assume that one or two drugs can be developed to potentially cure a disease. If one or two drugs cannot be developed, the model reduces to the special case where the one or two drugs are not available. Third, we assume that disease recovery is possible to some extent with or without one or two drugs. We allow for great variation in the degree of recovery, from complete recovery, via some recovery, no recovery, and death.

The model’s assumptions abstracts away healthcare workers, hospitals, governments, international organizations, and various other players in our health and political/economical systems. These other players are crucial, e.g. as advisors, facilitators, and providers of knowledge and services. By abstracting away these other players and factors, we are able to focus explicitly on the strategic interaction between the N persons, the pharmaceutical company, the donor (which is parametric in the analysis), and Nature.

The model helps understand individual behavior regarding contracting disease and the purchasing of drugs when interacting with a pharmaceutical company which may or may not develop drugs, a donor which may or may not subsidize, and Nature which may precariously determine disease contraction, recovery, death, and virus mutation. The model is integrated in the sense that it brings the relevant players together in the decision making process.

Empirical data is provided of HIV/AIDS data for prevalence, deaths, HIV expenditure, treatment costs, R&D costs and revenues, and HIV resource availability are presented. In 2019, 38 million people lived with HIV/AIDS.^1^ Using the data, a procedure is presented for estimating the model parameters. An example shows how the players’ strategic decisions may cause various outcomes.

### The literature

Game theoretic analysis of interaction between persons and a pharmaceutical company is uncommon. Four game theoretic studies have been identified. Hausken and Ncube [18, 19] analyze interactions between policy makers choosing resource allocation between prevention and treatment of disease, the international community choosing funding to treat disease, and Nature choosing which proportion of the population contracts disease, and which fractions remains sick or does, versus recovers. Mamani, Chick, and Simchi-Levi [29] develop a game theoretic model of international influenza vaccination coordination. Hausken and Ncube [20] consider a game between a drug company and patients which contemplate whether to purchase a drug.

Aside from these four studies, the literature is more tangential to this article. A literature does exist on disease treatment and prevention. Below we review this literature, and attempt to explain or justify their interest for this article, which is implicitly focused on disease treatment and prevention. The literature focuses strongly on treatment rather than prevention, in contrast to this article which focuses on how drugs may or may not be developed and funded as a consequence of a disease being contracted or not contracted. For example, Thomas [35], Kremer and Glennerster [25], and Kremer and Snyder [26, 27] suggest that incentives for drug development for treatment outweigh incentives for vaccine development for prevention. Potentially, with such a focus more citizens may become sick, causing more resources to treatment than prevention.

With the strong focus on treatment in the literature, Hecht et al. [21] and Izazola-Licea et al. [24] assess the financing of the response to HIV/AIDS in low-income and middle-income countries. West and Schneider [38] estimate revenues for HIV/AIDS treatment for the years 2017–2021 for various African countries. Forsythe et al. [13] assess the global costs, health achievements, and economic benefits of 20 years of ART (antiretroviral therapy) for people living with HIV. DiMasi et al. [11] stipulate $2.6 billion for HIV drug R&D costs for the years 2017–2021. Coates, Richter, and Caceres [8] evaluate behavioral strategies to reduce HIV transmission. Moxnes and Hausken [31] model acute virus influenza A infections.

For research on treatment versus prevention see Boily et al. [5], Bertozzi et al. [4], Canning [6], Alistar and Brandeau [1], Bärnighausen, Salomon, and Sangrujee [3], Gonsalves [16], Kumaranayake, Watts, Dixon, Mc Donald, and Roberts [28], and Paltiel and Stinnett [32], and the HIV Modelling Consortium Treatment as Prevention Editorial Writing Group [22]. Regarding the cost effectiveness of treatment and prevention, see Creese, Floyd, Alban, and Guinness [10], Granich et al. [17], Galárraga, Colchero, Wamai, and Bertozzi [14]. For the cost-effectiveness of prevention, see Goldie et al. [15], Cohen, Shin-Yi, and Farley [9], Walker [37], Hogan, Baltussen, Hayashi, Lauer, and Salomon [23],. Fitzpatrick, Singer, Hotez, and Galvani [12] recommend a Congressional cost-effectiveness committee to reveal underinvestment in public health compared with other sectors, and advance societal welfare and.

Section 2 provides the methods. Section 3 presents the theoretical results. Section 4 presents the empirical results. Section 5 recommends and exemplifies a procedure for estimating the model parameters. Section 6 discusses scope, limitations of the study, and future research. Section 7 concludes.

## Methods

### Nomenclature

#### Parameters

N Number of persons

G Number of persons choosing safe behavior

L Number of persons choosing risky behavior while not contracting the disease

M_j_ Number of persons not buying drug j, j = 1,2, with or without drug production by pharmaceutical company

m_1_ Number of persons buying drug 1

m_12_ Number of persons buying both drugs 1 and 2

C_j_ Drug j purchasing cost for for person i, j = 1,2; i = 1,…,N

c_j_ Drug j production cost for the pharmaceutical company destined for person i, j = 1,2; i = 1,…,N

k_j_ Exponential parameter scaling drug j production cost, j = 1,2

d_j_ Drug j development cost, j = 1,2

E_i_ Person i’s utility of risky behavior, i = 1,…,N

H_i_ Person i’s utility of safe behavior, H_i_ < E_i_, i = 1,…,N

R_i_ Person i’s utility when recovering from disease, R_i_ < H_i_, i = 1,…,N

D_i_ Person i’s utility of death, D_i_ < R_i_, i = 1,…,N

#### Strategic choices by person i, i = 1,…,N

Choice between risky behavior and safe behavior in period 1

Choice whether to buy drug 1 or not buy drug 1 in period 4

Choice whether to buy drug 2 or not buy drug 2 in period 7

#### Strategic choices by pharmaceutical company

Choice whether to develop drug 1 at cost d_1_ in period 3

Choice whether to develop drug 2 at cost d_2_ in period 6

#### Strategic choices by donor

X_1_ Subsidy fraction of drug 1 development cost d_1_ in period 3

S_1_ Subsidy fraction of drug 1 purchasing cost C_1_ for person i in period 4

X_2_ Subsidy fraction of drug 2 development cost d_2_ in period 6

S_2_ Subsidy fraction of drug 2 purchasing cost C_2_ for person i in period 7

#### Strategic choices by Nature

q Disease contraction probability in period 2

x Disease recovery probability without drug 1 in periods 4 and/or 5

r Virus mutation probability in period 5

w Disease recovery probability with drug 1 in period 6

v Disease recovery probability without drug 2 in periods 7 and/or 8

s Disease recovery probability with drug 2 in period 8

#### Dependent variables

p Fraction of the N persons choosing risky behavior

U_i_ Person i’s expected utility, i = 1,…,N

u Pharmaceutical company’s expected profit

V Donor’s expected utility

### The model

**Fig. 1:**
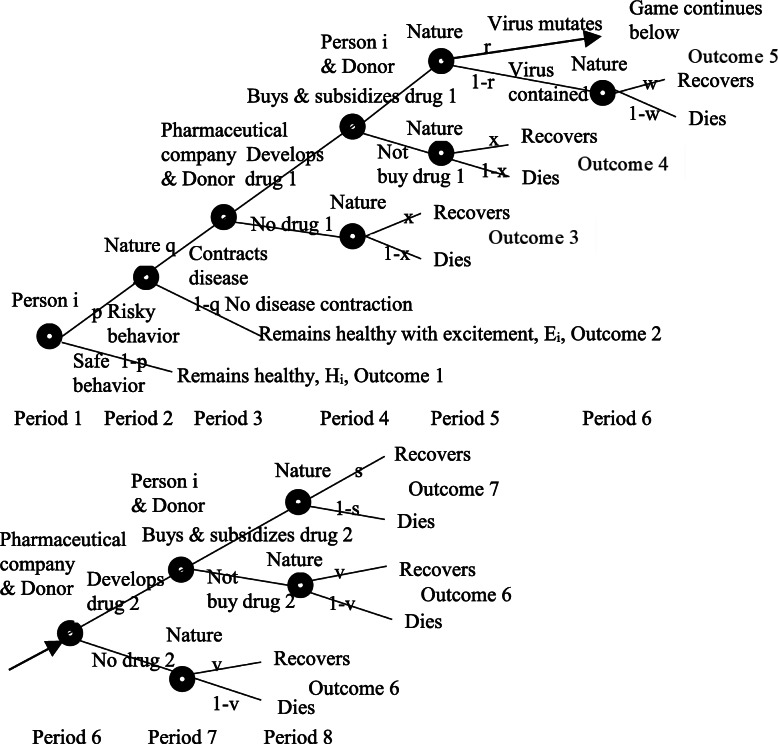
Eight-period game for person i, the pharmaceutical company, the donor, and Nature

We consider a population of N persons and the complete information eight-period game in Fig. 1 with four players and 13 choice (decision) nodes. The eight periods have been designed to reflect the natural flow of strategic choices by the players. The game naturally starts in period 1 with person i choosing risky or safe behavior. Also naturally, in period 2 Nature chooses whether risky behavior causes disease contraction. Intuitively, with the presence of disease contraction, in period 3 the pharmaceutical company needs to determine whether to develop drug 1. Consequently, if drug 1 is developed, in period 4 person i and the donor need to determine whether to buy and subsidize drug 1. Given the presence of drug 1 in some of the N persons, the natural next step, by Nature in period 5, is whether the virus mutates. If the virus mutates, again the pharmaceutical company needs to make a strategic choice, in period 6, i.e. whether to develop drug 2, since drug 1 is no longer operational after the mutation. If drug 2 is developed in period 6, that naturally has similar consequences as after drug 1 was developed in period 3. That is, in period 7 person i and the donor determine whether to buy and subsidize drug 2. Finally, in period 8 Nature chooses whether person i recovers or dies.

Person i has three choice nodes. The two last of these nodes are influenced by the donor (which may consist of multiple donors considered as one collective unit) subsidizing the costs C_1_ and C_2_ of drugs 1 and 2 with a fraction S_j_ for drug j, 0 ≤ S_j_ ≤ 1, j = 1,2, so that person i pays the remaining fraction 1-S_j_. The pharmaceutical company has two choice nodes, influenced by the donor subsidizing the costs d_1_ and d_2_ of the development of drugs 1 and 2 with a fraction X_j_ for drug j, 0 ≤ X_j_ ≤ 1, j = 1,2, so that the pharmaceutical company pays the remaining fraction 1-X_j_. Nature has eight choice nodes and six strategic choices. To ensure tractability all the N persons start the game at the same time and proceed through the eight periods at the same pace. Each person i prefers safe versus risky behavior differently, and assigns different utilities to remaining healthy without or with excitement, to death, and to recovery. The game may end after each period. The game generally ends in different periods for each person i depending on the different strategic choices.

Person i decides in period 1 whether to engage in risky or safe behavior. Risky behavior gives positive utility E_i_ if not contracting the disease. Safe behavior gives positive utility H_i_ < E_i_ which means remaining healthy. A fraction p and hence pN persons choose risky behavior, where p follows from which of the N persons choose risky behavior. Nature chooses in period 2 that risky behavior causes disease contraction with probability q. Thus pqN persons contract the disease. The pharmaceutical company chooses in period 3 either to develop drug 1 at cost d_1_ subsidized with a fraction X_1_, or to develop no drug at no cost. With no drug, Nature chooses in period 4 recovery with probability x and positive utility R_i_ < H_i_ for person i, or death with probability 1-x and negative utility D_i_ < R_i_ for person i, and the game ends. If drug 1 is developed, person i chooses in period 4 either not to buy it (causing Nature to choose recovery or death with the same probabilities x and 1-x as if the drug were not developed), or to buy it at cost C_1_ subsidized by donors with a fraction S_1_. If drug 1 is bought, in period 5 the virus is contained with probability 1-r (and Nature chooses recovery vs death in period 6) or the virus mutates with probability r. In the latter event the pharmaceutical company chooses in period 6 either to develop drug 2 at cost d_2_ subsidized with a fraction X_2_, or to develop no drug at no cost. With no drug, Nature chooses in period 7 recovery or death and the game ends. If drug 2 is developed, person i chooses in period 7 either not to buy it (causing Nature to choose recovery or death in period 8), or to buy it at cost C_2_ subsidized by donors with a fraction S_2_. If drug 2 is bought, in the final period 8 Nature chooses recovery vs death with probabilities s and 1-s respectively.

Summing up, person i has three strategic choice variables. It chooses risky or safe behavior in period 1, chooses whether or not to buy drug 1 in period 4 (if the pharmaceutical company has developed it in period 3), and chooses whether or not to buy drug 2 in period 7 (if the pharmaceutical company has developed it in period 6). The pharmaceutical company has two strategic choice variables, i.e. whether or not to develop drug 1 in period 3, and whether or not to develop drug 2 in period 6. The donor has four strategic choice variables. It chooses the subsidy fraction X_1_ to pay for drug 1 development in period 3, the subsidy fraction S_1_ to pay for each person i’s drug 1 purchase in period 4, the subsidy fraction X_2_ to pay for drug 2 development in period 6, and the subsidy fraction S_2_ to pay for each person i’s drug 2 purchase in period 7. Nature has six strategic choice variables in eight choice nodes. It chooses the disease contraction probability q in period 2, the recovery probability x in period 4 if drug 1 is not developed, the same recovery probability x in period 5 if drug 1 is developed but not bought (and thus not applied), the virus mutation probability r in period 5, the recovery probability w in period 6 if drug 1 is bought (and applied) and the virus is contained, the recovery probability v in period 7 if drug 2 is not developed, the same recovery probability v in period 8 if drug 2 is developed but not bought (and thus not applied), and the recovery probability s in period 8 if drug 2 is bought (and applied).

### Individual persons’ behavior, benefits, and costs

Figure 1 has seven outcomes when not counting Nature’s probabilistic choice of recovery or death. Counting these seven outcomes from the root in the game tree and outwards through the branches, and listing them from condition 1 through condition 7, person i’s expected utility is

1 $$ {U}_i=\left\{\begin{array}{c}{H}_i\kern0.5em if\kern0.5em safe\kern0.5em behavior\kern27.5em \\ {}{E}_i\kern0.5em if\kern0.5em risky\kern0.5em behavior\kern0.5em and\kern0.5em no\kern0.5em disease\kern0.5em contraction\kern17em \\ {}\left(1-x\right){D}_i+{xR}_i\kern0.5em if\kern0.5em risky\kern0.5em behavior\& disease\kern0.5em contraction\& no\kern0.5em drug\kern0.5em 1\kern0.5em development\kern3.5em \\ {}\begin{array}{l}\left(1-x\right){D}_i+{xR}_i\kern0.5em if\kern0.5em risky\kern0.5em behavior\& disease\kern0.5em contraction\& drug\kern0.5em 1\kern0.5em development\kern0.5em only\kern3em \\ {}\kern1em \& no t\kern0.5em buy\kern0.5em drug\kern0.5em 1\end{array}\\ {}\begin{array}{l}\left(1-r\right)\left[\left(1-w\right){D}_i+{wR}_i\right]+r\left[\left(1-v\right){D}_i+{vR}_i\right]-\left(1-{S}_1\right){C}_1\kern0.5em if\kern0.5em risky\kern0.5em behavior\& disease\kern3em \\ {}\kern1em contraction\& drug\kern0.5em 1\kern0.5em development\kern0.5em only\& buy\kern0.5em drug\kern0.5em 1\kern0.5em only\end{array}\\ {}\begin{array}{l}\left(1-v\right){D}_i+{vR}_i-\left(1-{S}_1\right){C}_1\kern0.5em if\kern0.5em risky\kern0.5em behavior\& disease\kern0.5em contraction\& drug\kern0.5em 1\kern0.5em development\\ {}\& buy\kern0.5em drug\kern0.5em 1\kern0.5em \& virus\kern0.5em mutation\& no t\kern0.5em buy\kern0.5em drug\kern0.5em 2\end{array}\\ {}\begin{array}{l}\left(1-s\right){D}_i+{sR}_i-\left(1-{S}_1\right){C}_1-\left(1-{S}_2\right){C}_2\kern0.5em if\kern0.5em risky\kern0.5em behavior\& disease\kern0.5em contraction\& drug\kern0.5em 1\kern1em \\ {}\kern1em development\& buy\kern0.5em drug\kern0.5em 1\kern0.5em \& virus\kern0.5em mutation\& drug\kern0.5em 2\kern0.5em development\& buy\kern0.5em drug\kern0.5em 2\end{array}\end{array}\right. $$

Equation (1) contains four parameters H_i_, E_i_, D_i_, R_i_ which differ for each person i, i = 1,…,N, two strategic choice variables (which are parametric in the analysis) S_1_ and S_2_ chosen by the donor which are equivalent for the N persons, five strategic choice variables (drawn by a random generator) x,r,w,v,s chosen by Nature, and which also are equivalent for the N persons, and the N persons’ drug purchasing costs C_1_ and C_2_. Nature’s strategic choice q of the disease contraction probability in period 2 is implicitly present in (1) since it impacts which of the seven conditions emerges. Which of the seven conditions in (1) emerges depends on the pharmaceutical company’s two strategic choices of whether to develop drug 1 in period 3 and develop drug 2 in period 6. Which of the seven conditions in (1) emerges also depends on person i’s three strategic choices, i.e. whether to choose risky or safe behavior in period 1, whether or not to buy drug 1 in period 4 (if available), and whether or not to buy drug 2 in period 7 (if available). Equation (1) may be defined as a distribution function in the sense that Nature’s five strategic choice variables x,r,w,v,s, drawn by a random generator, causes distribution across the outcomes if drawing from the random generator is done repeatedly. All parameters are common knowledge for all the players.

Condition 1 in (1) is valid for a risk averse person i which assigns high utility H_i_ to safe behavior and low expected utility to the outcomes of risky behavior when assessing the probabilities, drug production and costs. Condition 2 is valid when Nature chooses no disease contraction, that is q = 0, which gives utility E_i_ > H_i_. Conditions 3 and 4 in (1) are equivalent since if person i does not buy drug 1, then it is irrelevant whether drug 1 is developed or not. Condition 5 states that person i buys drug 1 at cost (1-S_1_)C_1_ after which the virus is contained with probability 1-r and mutates with probability r. Condition 6 states that the virus mutates which is a precondition for drug 2 development, and that person i does not buy drug 2. Outcome 6 appears twice in Fig. 1 since the pharmaceutical company may or may not develop drug 2. The final condition 7 states that drug 2 is developed and that person i buys it at cost (1-S_2_)C_2_.

Overall, with no drug development, only the first three outcomes in (1) are possible. The third outcome gives death with probability 1-x and recovery with probability x. Hence all four utilities H_i_,E_i_,D_i_,R_i_ are possible. If the pharmaceutical company develops drug 1 but not drug 2, the first five outcomes are possible. If the pharmaceutical company develops both drugs 1 and 2, all the seven outcomes are possible. The N persons have different H_i_,E_i_,D_i_,R_i_, and thus distribute themselves across the three or five or seven outcomes, depending on no drug development, development of drug 1 but not drug 2, and development of both drugs, respectively.

### Pharmaceutical company’s behavior, benefits, and costs

The expected profit of the pharmaceutical company depends on the numbers of persons buying drugs 1 and 2, the drug prices, and the costs of drug development. We assume that m_1_ persons buy drug 1 (and may or may not buy drug 2) which allows outcomes 5–7, and that a weakly smaller number m_12_, m_12_ ≤ m_1_ persons buy both drugs 1 and 2, which allows only outcome 7. This assumption is in accordance with Fig. 1 where persons are not allowed to enter the game in later periods. Hence m_1_-m_12_ persons buy only drug 1 which allows outcome 5. Furthermore, assuming that person i has bought drug 1, in order to buy drug 2 the virus has to mutate, and the pharmaceutical company must develop drug 2. The pharmaceutical company’s expected profit is

2 $$ u=\left\{\begin{array}{l}0\kern0.5em if\kern0.5em no\kern0.5em drug\kern0.5em development\\ {}{m}_1{C}_1-{\left({m}_1{c}_1\right)}^{k_1}-\left(1-{X}_1\right){d}_1\kern0.5em if\kern0.5em only\kern0.5em drug\kern0.5em 1\kern0.5em development\\ {}{m}_1{C}_1+{m}_{12}{C}_2-{\left({m}_1{c}_1\right)}^{k_1}-{\left({m}_{12}{c}_2\right)}^{k_2}-\left(1-{X}_1\right){d}_1-\left(1-{X}_2\right){d}_2\\ {} if\kern0.5em r=1\kern0.5em \&\kern0.5em drug\kern0.5em 1\kern0.5em \&\kern0.5em drug\kern0.5em 2\kern0.5em development\end{array}\right. $$

where k_j_ scales the production cost for drug j, and c_j_ is the drug production cost for drug j, j = 1,2, for the pharmaceutical company in the amount required for person i, i = 1,…,N. Equation (2) contains two strategic choice variables (which are parametric) X_1_ and X_2_ chosen by the donor, one strategic choice variable (drawn by a random generator) r chosen by Nature, and the nine parameters m_1_,c_1_,k_1_,d_1_,m_12_,k_2_,d_2_,C_1_,C_2_. Which of the four conditions in (2) emerges depends on the pharmaceutical company’s two strategic choices of whether to develop drug 1 in period 3 and develop drug 2 in period 6, and depends on the strategic choices by the N persons, the donor, and Nature.

When k_j_ = 1, production cost is linear. When 0 < k_j_ < 1, production cost is concave (economy of scale). When k_j_ > 1, production cost is convex (diseconomy of scale). Condition 1 in (2) corresponds to m_1_ = m_12_ = 0 where the N persons distribute themselves across outcomes 1–3, condition 2 corresponds to m_1_ ≥ 0 = m_12_ where the N persons distribute themselves across outcomes 1–5, and condition 3 presumes virus mutation r = 1 and corresponds to m_1_ > 0 and m_12_ ≥ 0 where the N persons distribute themselves across all seven outcomes.

Although person i has three choice nodes and the pharmaceutical company has two choice nodes, four of these five choice nodes are not reached if all N persons choose safe behavior (outcome 1), or if those that choose risky behavior do not contract the disease (outcome 2). For the pharmaceutical company to develop drug 1 in its first node, at least one person must contract the disease and then that person can choose whether or not to buy drug 1. For the pharmaceutical company to develop drug 2 in its second node, the virus must mutate and then persons can choose whether or not to buy drug 2.

Table 1 lists the seven outcomes in the first row, and the number of persons choosing each outcome in row 2, where G is the number of persons choosing safe behavior and L (for lucky) is the number of persons choosing risky behavior while not contracting the disease. We define M_j_ as the number of persons not buying drug j despite drug j being produced by the pharmaceutical company, j = 1,2. With these definitions, N-G-L-M_1_-m_1_ is the unfortunate number of persons with outcome 3 contracting the disease while no drugs are available. Row 3 lists person i’s strategy. Of interest to the pharmaceutical company are row 4 showing the number m_1_ of persons buying drug 1 and row 5 showing the number m_12_ of persons buying both drugs 1 and 2. Drug 2 is developed only if the virus mutates. Row 6 shows that the pharmaceutical company earns zero or negative expected profit for outcomes 1–4, and may earn positive expected profit for outcomes 5–7. The bottom row 7 shows the pharmaceutical company’s drug development strategies.

**Table 1:** Outcomes, number of persons choosing the various strategies, and the pharmaceutical company’s drug development strategies

Outcome	1	2	3	4	5	6	7
Number of persons	G	L	N-G-L-M_1_-m_1_	M_1_	m_1_-M_2_- m_12_	M_2_	m_12_
Person i	Safe behavior	Risky behavior	Disease contraction	Not buy drug 1	Buy drug 1	Not buy drug 2	Buy drug 2
m_1_	0	0	0	0	> 0	> 0	> 0
m_12_	0	0	0	0	0	0	> 0
Company’s expected profit	0	0	0	−(1 − X_1_)d_1_	Eq (2)	Eq (2)	Eq (2)
Company	No drug development			Drug 1 development		Drug 2 development	

In summary, the pharmaceutical company does not develop drugs in outcomes 1–3, where expected profits are zero. The pharmaceutical company does develop drug 1 in outcomes 4 and 5, and subsequently develops drug 2 in outcomes 6 and 7.

### Donor’s behavior, benefits, and costs

We assume that the donor’s benefit is the sum of the N persons’ benefits H_i_,E_i_,D_i_,R_i_ accounting for the probabilities q,r,s,v,w,x, strategies, and numbers of persons choosing the seven outcomes. The donor subsidizes the development cost of drug j with X_j_d_j_, where X_j_ is the donor subsidy fraction, j = 1,2. The donor subsidizes the drug purchasing cost of drug j for person i with S_j_C_j_, where S_j_ is the donor subsidy fraction, j = 1,2; i = 1,…,N. Since m_1_ persons purchase drug 1, the donor subsidizes drug 1 with m_1_S_1_C_1_. Since m_12_ persons purchase drug 2, the donor subsidizes drug 2 with m_2_S_2_C_2_. The subsidy fractions S_j_ and X_j_ are assumed to be parametric and thus the strategic choices of the donor are not considered. For the donor’s expected utility we get the same three conditions as for the pharmaceutical company’s expected profit in (2), i.e.

3 $$ V=\left\{\begin{array}{c}\sum \limits_{i=1}^G{H}_i+\sum \limits_{i=G+1}^{G+L}{E}_i+\sum \limits_{i=G+L+1}^{N-{M}_1-{m}_1}\left[\left(1-x\right){D}_i+{xR}_i\right]\kern0.5em and\kern0.5em {M}_1={m}_1=0\kern0.5em if\kern0.5em no\kern0.5em drug\kern0.5em development\\ {}\begin{array}{l}\sum \limits_{i=1}^G{H}_i+\sum \limits_{i=G+1}^{G+L}{E}_i+\sum \limits_{i=G+L+1}^{N-{M}_1-{m}_1}\left[\left(1-x\right){D}_i+{xR}_i\right]+\sum \limits_{i=N-{M}_1-{m}_1+1}^{N-{m}_1}\left[\left(1-x\right){D}_i+{xR}_i\right]\kern4.5em \\ {}\kern1em +\sum \limits_{i=N-{m}_1+1}^{N-{M}_2-{m}_{12}}\left(\left(1-r\right)\left[\left(1-w\right){D}_i+{wR}_i\right]+r\left[\left(1-v\right){D}_i+{vR}_i\right]-{S}_1{C}_1\right)\\ {}\kern1em -{X}_1{d}_1\kern0.5em and\kern0.5em {M}_2={m}_{12}=0\kern0.5em if\kern0.5em only\kern0.5em drug\kern0.5em 1\kern0.5em development\end{array}\\ {}\begin{array}{l}\sum \limits_{i=1}^G{H}_i+\sum \limits_{i=G+1}^{G+L}{E}_i+\sum \limits_{i=G+L+1}^{N-{M}_1-{m}_1}\left[\left(1-x\right){D}_i+{xR}_i\right]+\sum \limits_{i=N-{M}_1-{m}_1+1}^{N-{m}_1}\left[\left(1-x\right){D}_i+{xR}_i\right]\\ {}\kern1em +\sum \limits_{i=N-{m}_1+1}^{N-{M}_2-{m}_{12}}\left(\left(1-v\right){D}_i+{vR}_i-{S}_1{C}_1\right)+\sum \limits_{i=N-{M}_2-{m}_{12}+1}^{N-{m}_{12}}\left(\left(1-v\right){D}_i+{vR}_i-{S}_1{C}_1\right)\kern3em \\ {}\kern1em +\sum \limits_{i=N-{m}_{12}+1}^N\left(\left(1-s\right){D}_i+{sR}_i-{S}_1{C}_1-{S}_2{C}_2\right)\kern0.5em -{X}_1{d}_1-{X}_2{d}_2\\ {}\kern1em if\kern0.5em r=1\kern0.5em \&\kern0.5em drug\kern0.5em 1\kern0.5em \&\kern0.5em drug\kern0.5em 2\kern0.5em development\end{array}\end{array}\right. $$

The first condition in (3) covers outcomes 1–3 with no subsidies. The second condition covers outcomes 1–5 with subsidies S_1_C_1_ and X_1_d_1_. The third condition covers outcomes 1–7 with subsidies S_j_C_j_ and X_j_d_j_, j = 1,2. Equation (3) contains four parameters H_i_, E_i_, D_i_, R_i_ which differ for each person i, i = 1,…,N, four strategic choice variables (which are parametric) S_1_,X_1_,S_2_,X_2_ chosen by the donor which are equivalent for the N persons, five strategic choice variables (drawn by a random generator) x,r,w,v,s chosen by Nature, and the nine parameters G,L,N,M_1_,m_1_,M_2_,m_12_,C_1_,C_2_. Which of the three conditions in (3) emerges depends on whether no drug is developed, only drug 1 is developed, and both drugs 1 and 2 are developed. Healthcare workers are not explicitly present in Fig. 1, but play a role by advising persons on risky versus safe behavior and whether or not to buy the drugs, and advising the pharmaceutical about the attributes of the disease and other factors relevant for drug development.

## Theoretical results

We solve the game with backward induction starting with period 8. Nature’s strategies are probabilistic with the probabilities in Fig. 1. Hence we start with period 7 where person i buys drug 2 if the benefits from buying is at least as large as not buying it, that is, if

4 $$ {\displaystyle \begin{array}{l}\left(1-s\right){D}_i+{sR}_i-\left(1-{S}_1\right){C}_1-\left(1-{S}_2\right){C}_2\ge \left(1-v\right){D}_i+{vR}_i-\left(1-{S}_1\right){C}_1\\ {}\Rightarrow \left(s-v\right)\left({R}_i-{D}_i\right)\ge \left(1-{S}_2\right){C}_2\end{array}} $$

If (4) is not satisfied for m_12_ persons, the pharmaceutical company does not develop drug 2. Otherwise in period 6 the pharmaceutical company develops drug 2 if the expected profit from developing drug 2 outweigh those of not developing drug 2, that is, if

5 $$ {m}_1{C}_1+{m}_{12}{C}_2-{\left({m}_1{c}_1\right)}^{k_1}-{\left({m}_{12}{c}_2\right)}^{k_2}-\left(1-{X}_1\right){d}_1-\left(1-{X}_2\right){d}_2\ge {m}_1{C}_1-{\left({m}_1{c}_1\right)}^{k_1}-\left(1-{X}_1\right){d}_1\Rightarrow {m}_{12}{C}_2-{\left({m}_{12}{c}_2\right)}^{k_2}\ge \left(1-{X}_2\right){d}_2 $$

In period 4 person i buys drug 1 if the benefits of consuming drug 1 outweigh the benefits of not consuming drug 1, that is, if

6 $$ {\displaystyle \begin{array}{l}\left(1-r\right)\left(\left(1-w\right){D}_i+{wR}_i\right)+r\left[\left(1-v\right){D}_i+{vR}_i\right]-\left(1-{S}_1\right){C}_1\ge \left(1-x\right){D}_i+{xR}_i\\ {}\Rightarrow \left(w-x+r\left(v-w\right)\right)\left({R}_i-{D}_i\right)\ge \left(1-{S}_1\right){C}_1\end{array}} $$

If (6) is not satisfied for m_1_ persons, the pharmaceutical company does not develop drug 1. Otherwise in period 3 the pharmaceutical company develops drug 1 if it is profitable to do so, that is, if

7 $$ {m}_1{C}_1-{\left({m}_1{c}_1\right)}^{k_1}-\left(1-{X}_1\right){d}_1\ge 0\Rightarrow {m}_1{C}_1-{\left({m}_1{c}_1\right)}^{k_1}\ge \left(1-{X}_1\right){d}_1 $$

In period 1 person i makes the following considerations, given that it does not know whether the virus mutates in period 5. If neither drug 1 nor drug 2 are optimal for it to buy, it chooses risky behavior if

8 $$ \left(1-q\right){E}_i+q\left(\left(1-x\right){D}_i+{xR}_i\right)\ge {H}_i $$

If drug 1 is optimal for it to buy whereas drug 2 is not optimal for it to buy, it chooses risky behavior if

9 $$ \left(1-q\right){E}_i+q\left(\left(1-r\right)\left(\left(1-w\right){D}_i+{wR}_i\right)+r\left[\left(1-v\right){D}_i+{vR}_i\right]-\left(1-{S}_1\right){C}_1\right)\ge {H}_i $$

If both drugs 1 and 2 are optimal for it to buy, it chooses risky behavior if

10 $$ \left(1-q\right){E}_i+q\left(\left(1-r\right)\left(\left(1-w\right){D}_i+{wR}_i\right)+r\left[\left(1-s\right){D}_i+{sR}_i-\left(1-{S}_2\right){C}_2\right]-\left(1-{S}_1\right){C}_1\right)\ge {H}_i $$

With no disease contraction, inserting q = 0 into (8), (9), or (10) gives

11 $$ {E}_i\ge {H}_i $$

which is satisfied by assumption guaranteeing risky behavior. Outcome 1 means that person i prefers safe behavior, which occurs if neither (8) nor (9) nor (10) are satisfied. Table 2 lists the seven outcomes O in the left column, lists the equations that apply in the middle column, and lists the conditions in the right column.

**Table 2:** The seven outcomes O, the equations that apply, and an example (section 5, i.e. the section labelled "Estimating and exemplifying the model parameters"). Yes and No in the rightmost column specify whether each example inequality matches whether the inequality in the corresponding equation should be satisfied

O	Description	Equations	Conditions
1	Person i prefers safe behavior	Neither (8) nor (9) nor (10) are satisfied.	(8),272,000 < 500,000 Yes (9),559,995 > 500,000 No (10),847,993 > 500,000 No
2	No disease contraction, causing risky behavior	(8) or (9) or (10) is satisfied.	(8),272,000 < 500,000 No (9),559,995 > 500,000 Yes (10),847,993 > 500,000 Yes
3	Contracting disease and no drug development	(4),(5),(6),(7) are not satisfied, and (8) is satisfied	(4),5.76 × 10^6^ > 50 No (5),1.4 × 10^9^ > 1.3 × 10^9^ No (6),2.88 × 10^6^ > 50 No (7),2.0 × 10^9^ > 1.3 × 10^9^ No (8),272,000 < 500,000 No
4	Contracting disease, drug development, but not buying drug 1	(4),(5),(6) are not satisfied, (7) and (8) are satisfied	(4),5.76 × 10^6^ > 50 No (5),1.4 × 10^9^ > 1.3 × 10^9^ No (6),2.88 × 10^6^ > 50 No (7),2.0 × 10^9^ > 1.3 × 10^9^ Yes (8),272,000 < 500,000 No
5	Contracting disease, buying drug 1, virus is contained, and no development of drug 2	(4) and (5) are not satisfied, (6),(7),(9) are satisfied	(4),5.76 × 10^6^ > 50 No (5),1.4 × 10^9^ > 1.3 × 10^9^ No (6),2.88 × 10^6^ > 50 Yes (7),2.0 × 10^9^ > 1.3 × 10^9^ Yes (9),559,995 > 500,000 Yes
6	Contracting disease, buying drug 1, virus mutation, and not buying drug 2 (which may or may not be developed)	(4) is not satisfied, (5),(6),(7),(9) are satisfied	(4),5.76 × 10^6^ > 50 No (5),1.4 × 10^9^ > 1.3 × 10^9^ Yes (6),2.88 × 10^6^ > 50 Yes (7),2.0 × 10^9^ > 1.3 × 10^9^ Yes (9),559,995 > 500,000 Yes
7	Contracting disease and buying drugs 1 and 2	(4),(5),(6),(7),(10) are satisfied	(4),5.76 × 10^6^ > 50 Yes (5),1.4 × 10^9^ > 1.3 × 10^9^ Yes (6),2.88 × 10^6^ > 50 Yes (7),2.0 × 10^9^ > 1.3 × 10^9^ Yes (10),847,993 > 500,000 Yes

## Empirical results

Between 2000 and 2007, the median price for first-line therapy in developing countries fell from $10,000 to below $100 per patient per year, which approximately is also the price today [30]. In 2017 the Clinton Health Access Initiative [7] and partners announced an agreement to produce a single HIV pill HIV to public sector purchasers in low- and middle-income countries to around $75 per person per year. Pillai et al. [33] find that the mean yearly cost of pre-ART HIV care is $158.52.

Many “People Living With HIV/AIDS” require second-line treatment due to resistance to first-line drug treatment or not tolerating first-line drugs. The World Health Organization [40] announces in 2007 that the median price for the most frequently used first- and second-line HAART (abacavir + didanosine + lopinavir/ritonavir) treatment for low-income countries was $1214. The World Health Organization [40] announces that for 2008, in middle-income countries, the price for second-line therapy was $3306 per year, as compared to $91 for first-line therapy. Médecins Sans Frontières [30] announces $87 as the cheapest first-line price and $749 (tenofovir + emtricitabine + lopinavir/ritonavir) as the cheapest second-line price.

Companies such as GlaxoSmithKline, Merck, Bristol Myers Squibb, offering ARV, adjust prices depending on the countries’ socioeconomic status, applying their own categorizations [30] or categorizations developed by the World Bank [39]. Prices are also determined by acquisition processes and third party negotiation [7]. Also, as expected, The World Health Organization [40] announces that large-scale production causes lower process.

During 2017–2021 DiMasi et al. [11] estimate $2.6 billion in HIV drug R&D costs, and West and Schneider [38] estimate $2.2 billion in revenues for South Africa, Nigeria, Tanzania, Ethiopia, the Democratic Republic of the Congo, and Egypt; $4.3 billion in revenues for the Middle East and North Africa and Sub-Saharan Africa; and $6.1 billion in worldwide revenues.

Table 3 shows in row 1 the HIV resource availability in US$ billion for low- and middle-income countries in 2018, and as percentages in row 2.

**Table 3:** Total HIV resource availability in US$ billion for low- and middle-income countries in 2018, and as percentages of the sum

Year	Domestic (Public and Private)	Global Fund	United States (bilateral)	Other international	Sum
2018	10,659.15	1600.24	5139.08	1620.89	19,019.36
2018%	56.04	8.41	27.02	8.52	100.00

## Estimating and exemplifying the model parameters

Let us estimate the model’s parameters, including the donor’s strategic choices X_1_,S_1_,X_2_,S_2_ and Nature’s strategic choices q,x,r,w,v,s, see the nomenclature in the beginning of the Methods section. The drug purchasing cost C_j_ for drug j, j = 1,2, for person i, i = 1,…,N, is estimated as C_j_ = $100 per year, which falls within $75–$158.52 per year estimated in the previous section. The drug production cost c_j_ for drug j, j = 1,2, for the pharmaceutical company destined for person i, i = 1,…,N, is estimated as $80 per person per year, which is 20% below C_j_ = $100. The exponential parameter k_j_ scaling the drug production cost for drug j, j = 1,2, is estimated as k_j_ = 0.5, which assumes sufficiently large markets and efficient production (since k_j_ < 1, concave production, economy of scale). (That contrasts with k_j_ = 1 which would mean linear production and k_j_ > 1 which would mean convex production and diseconomy of scale.) The drug development cost d_j_ for drug j, j = 1,2, is in the previous section estimated to be d_j_ = $2.6 billion, where we due to simplicity assume equivalent costs of developing the two drugs.

Using Appelbaum’s [2] estimate $6.1–$9.1 million of the value of statistical life, person i’s utility D_i_ of death is estimated as D_i_ = −$7 million. (Such valuations typically depend on how a person strikes a balance between health risks and rewards, or on weighing wages against death risk in the labor market.) Person i’s utility E_i_ of risky behavior, i = 1,…,N, is estimated as 1/7 of the value of statistical life, i.e. E_i_ = 1 million. Person i’s utility H_i_ of safe behavior, H_i_ < E_i_, i = 1,…,N, is estimated as H_i_ = 0.5E_i_ = $0.5 million. Person i’s utility R_i_ when recovering from disease, D_i_ < R_i_ < H_i_, i = 1,…,N, is estimated as R_i_ = 0.4H_i_ = $0.2 million.

The subsidy fraction X_j_, j = 1,2, of drug development cost d_1_ in period 3 and d_2_ in period 6 is estimated intermediately as X_j_ = 0.5. (Since the pharmaceutical company is usually profit-seeking, the subsidy fraction X_j_ of the drug development cost is usually below one.) Similarly, the subsidy fraction S_j_, j = 1,2, of drug purchasing cost C_1_ for person i in period 4 and C_2_ for person i in period 7 is estimated intermediately as S_j_ = 0.5. (Countries with extensive social welfare programs may choose a higher subsidy fraction S_j_.) The estimates of X_j_ and S_j_ may be assessed further in view of the percentages in Table 3.

The disease contraction probability q in period 2 is estimated as q = 0.1, influenced by HIV/AIDS data from UNAIDS [36] which show the HIV prevalence fractions among adults aged 15–49. Although these prevalence fractions range from < 0.1 for many countries to 0.273 for Eswatini, q is usually higher than the prevalence. In fact, frequent risky behavior in interaction with persons who have contracted the disease, or in environments with high HIV prevalence, may cause q to be close to one, though with very low HIV prevalence it can be much lower. The disease recovery probability x without drug 1 in periods 4 and/or 5 is estimated as x = 0.1. It can be expected to be low, and close to or equal to zero for serious or deadly diseases. The virus mutation probability r in period 5 is estimated as r = 0.5. The disease recovery probability w with drug 1 in period 6 if the virus is contained is estimated as w = 0.9, thus assuming that drug 1 is useful. The disease recovery probability v without drug 2 in periods 7 and/or 8 is estimated as v = 0.1, assuming that refraining from using drug 2 is risky. The disease recovery probability s in period 8 if drug 2 is bought (and applied) is estimated as s = 0.9, thus assuming that drug 2 is useful.

The inequalities in the rightmost column in Table 2 follow from assuming the parameter values above, assuming that m_1_ = 20 million persons buy drug 1, and that m_12_ = 14 million persons buy drug 2. Outcome 1 does not follow since (9) and (10) are satisfied, while (8) is not satisfied, which induces risky behavior. If person i’s utility H_i_ = $0.5 million of safe behavior increases above H_i_ = $0.847993 million, then (10) is not satisfied, and person i chooses safe behavior instead. If person i chooses risky behavior, with subsequent disease contraction, outcomes 1 and 2 are impossible, and outcomes 3,4,5,6 or 7 arise instead. Outcome 2 with risky behavior is possible (with probability 1-q) since (8) or (9) or (10) is satisfied, and arises with probability 1-q chosen by Nature of no disease contraction. Outcome 3 does not follow since (4),(5),(6),(7) are satisfied, while (8) is not satisfied, and hence the pharmaceutical company proceeds to develop drug 1. The pharmaceutical company develops drug 1 if at least m_1_ = 14 million persons buy drug 1. Since m_1_ = 20 million, drug 1 is developed. Outcome 4 does not follow since (4),(5),(6),(7) are satisfied, while (8) is not satisfied. Hence the pharmaceutical company proceeds to develop drug 1, person i buys it, and the donor subsidizes it. Outcome 5 with death (probability 1-w) or recovery (probability w) is possible since (4), (5), (6), (7), (9) are satisfied, and arises with probability 1-r chosen by Nature of virus containment. Outcome 6 with death (probability 1-v) or recovery (probability v) is possible since (4), (5), (6), (7), (9) are satisfied, and arises with probability r chosen by Nature of virus mutation. Outcome 6 is possible if the pharmaceutical company does not develop drug 2, or the pharmaceutical company develops drug 2 but person i does not buy it. The pharmaceutical company develops drug 2 if at least m_12_ = 14 million persons buy drug 2, which is satisfied. Outcome 7 is the only row in Table 2 which specifies Yes to all the inequalities. That is, (4), (5), (6), (7), 10) are satisfied. Outcome 7 means that person i chooses risky behavior, contracts the disease, and buys both drugs 1 and 2, which means that the pharmaceutical company develops both drugs. Outcome 7 assumes that Nature chooses disease contraction with probability q and virus mutation with probability r. Outcome 7 means that person i dies with probability 1-s or recovers with probability s. Inserting the parameter values into (1), person i’s expected utility is

12 $$ {U}_i=\left\{\begin{array}{c}\$0.5\times {10}^6\kern0.5em if\kern0.5em safe\kern0.5em behavior\kern21.5em \\ {}\${10}^6\kern0.5em if\kern0.5em risky\kern0.5em behavior\kern0.5em and\kern0.5em no\kern0.5em disease\kern0.5em contraction\kern12em \\ {}-\$6.28\times {10}^6\kern0.5em if\kern0.5em risky\kern0.5em behavior\& disease\kern0.5em contraction\& no\kern0.5em drug\kern0.5em 1\kern0.5em development\kern1em \\ {}\begin{array}{l}-\$6.28\times {10}^6\kern0.5em if\kern0.5em risky\kern0.5em behavior\& disease\kern0.5em contraction\& drug\kern0.5em 1\kern0.5em development\kern0.5em only\\ {}\kern1em \& no t\kern0.5em buy\kern0.5em drug\kern0.5em 1\end{array}\\ {}\begin{array}{l}-\$3.40\times {10}^6\kern0.5em if\kern0.5em risky\kern0.5em behavior\& disease\kern0.5em contraction\& drug\kern0.5em 1\kern0.5em development\kern0.5em only\\ {}\kern1em \& buy\kern0.5em drug\kern0.5em 1\kern0.5em only\end{array}\\ {}\begin{array}{l}-\$6.28\times {10}^6\kern0.5em if\kern0.5em risky\kern0.5em behavior\& disease\kern0.5em contraction\& drug\kern0.5em 1\kern0.5em development\kern2em \\ {}\kern1.5em \& buy\kern0.5em drug\kern0.5em 1\kern0.5em \& virus\kern0.5em mutation\& no t\kern0.5em buy\kern0.5em drug\kern0.5em 2\end{array}\\ {}\begin{array}{l}-\$5.20\times {10}^5\kern0.5em if\kern0.5em risky\kern0.5em behavior\& disease\kern0.5em contraction\& drug\kern0.5em 1\kern0.5em development\kern2em \\ {}\kern1em \& buy\kern0.5em drug\kern0.5em 1\kern0.5em \& virus\kern0.5em mutation\& drug\kern0.5em 2\kern0.5em development\& buy\kern0.5em drug\kern0.5em 2\end{array}\end{array}\right. $$

In (12) person i prefers outcome 2 (line 2) if the disease is not contracted. If the disease is contracted after risky behavior, person i prefers outcome 5 if the virus is contained (line 5), or outcome 7 (line 7) if the virus mutates which gives the highest expected utility − 5.2 × 10^5^. Weighing outcome 2 and either outcome 5 or outcome 7 against each other with the given probabilities chosen by Nature, person i chooses risky behavior rather than safe behavior. Consequently, with these parameter values, the availability of both drugs induces risky behavior. Inserting the parameter values into (2), the pharmaceutical company’s expected profit is

13 $$ u=\left\{\begin{array}{c}0\kern0.5em if\kern0.5em no\kern0.5em drug\kern0.5em development\kern10em \\ {}\$7.00\times {10}^8\kern0.5em if\kern0.5em only\kern0.5em drug\kern0.5em 1\kern0.5em development\kern6.1em \\ {}\$8.00\times {10}^8\kern0.5em if\kern0.5em r=1\kern0.5em \&\kern0.5em drug\kern0.5em 1\kern0.5em \&\kern0.5em drug\kern0.5em 2\kern0.5em development\end{array}\right. $$

Hence the pharmaceutical company prefers to develop drug 1 if the virus is contained, develop both drugs if the virus mutates. The donor’s expected utility requires specifying the five additional parameters N,G,L,M_1_,M_2_. The number N of persons is assumed to be *N* = 100 million, which is the population one studies, e.g. a country in the data provided by UNAIDS UNAIDS [36], or chosen from a statistics database. The number G of persons choosing safe behavior is estimated to be G = 40 million. The number must evidently be less than N, and may be corroborated e.g. by purchases of protections against sexually transmitted diseases (e.g. condoms), or by other indicators of safe behavior. The number L of persons choosing risky behavior while not contracting the disease is estimated to be L = 30 million. It must evidently be less than N-G = 70 million. The number of m_1_ of persons buying drug 1 was estimated above to be m_1_ = 20 million. It must evidently be less than N-G-L = 30 million, since person i will not buy drug 1 without disease contraction. The number M_1_ of persons not buying drug 1 despite drug production by the pharmaceutical company is estimated to be M_1_ = 7 million. It must evidently be less than N-G-L-m_1_ = 10 million. The number of m_12_ of persons buying drug 2 was estimated above to be m_12_ = 14 million. It must evidently be less than N-G-L = 30 million, since person i will not buy drug 2 without disease contraction. The number M_2_ of persons not buying drug 2 despite drug production by the pharmaceutical company is estimated to be M_2_ = 4 million. It must evidently be less than N-G-L-m_12_ = 16 million. The above estimates implies that N-G-L-M_1_-m_1_ = 3 million persons contract the disease without drugs being available, since the pharmaceutical company does not develop drugs. We assume that all persons are equivalent to person i. Inserting these parameter values into (3), the donor’s expected utility is

14 $$ V=\left\{\begin{array}{c}-\$1.384\times {10}^{14}\kern0.5em and\kern0.5em {M}_1={m}_1=0\kern0.5em if\kern0.5em no\kern0.5em drug\kern0.5em development\kern2em \\ {}-\$8.080\times {10}^{13}\kern0.5em and\kern0.5em {M}_2={m}_{12}=0\kern0.5em if\kern0.5em only\kern0.5em drug\kern0.5em 1\kern0.5em development\\ {}-\$5.776\times {10}^{13}\kern0.5em if\kern0.5em r=1\kern0.5em \&\kern0.5em drug\kern0.5em 1\kern0.5em \&\kern0.5em drug\kern0.5em 2\kern0.5em development\kern3em \end{array}\right. $$

Equation (14) implies that the donor prefers the development of both drugs if the virus mutates, which gives the highest expected utility, and prefers that drug 1 is developed if the virus is contained. The lowest expected utility in line 1 in (14), with no drug development, follows from the third sum in (3) which sums the negative expected utility (1 − *x*)*D*_*i*_ + *xR*_*i*_ for person i over N-G-L persons, instead of summing over N-M_1_-m_1_-G-L persons as in line 2. The fourth sum in line 2 in (3) also sums the negative expected utility (1 − *x*)*D*_*i*_ + *xR*_*i*_ for person i, over M_1_ persons who do not buy drug 1. In contrast, the fifth sum in line 3 in (3) sums the expected utility ((1 − *r*)[(1 − *w*)*D*_*i*_ + *wR*_*i*_] + *r*[(1 − *v*)*D*_*i*_ + *vR*_*i*_] − *S*_1_*C*_1_) for person i, which is higher than (1 − *x*)*D*_*i*_ + *xR*_*i*_ since r = 0.5 and 0.1 = v = x ≤ w = 0.9, over m_1_ persons who buy drug 1. When m_1_ is sufficiently high, that causes line 2 in (14) to be higher than line 1. That all three lines in (14) are negative may be common for a donor. It may be due to factors not accounted for in this article, e.g. non-monetary rewards (e.g. a reputation for being altruistic), charitable contributions, or aid from governments or public or private enterprises which may seek economic welfare which may follow when the labor force is more healthy.

## Discussion

This section discusses the scope and implications of the study, highlights the limitations, and considers future research. The model’s scope involves strategic interaction between N persons choosing risky versus safe behavior, a pharmaceutical company choosing whether or not to develop one or two drugs depending on virus mutation, a donor assumed to be parametric, and Nature. The seven outcomes are that person i chooses safe behavior, chooses risky behavior without disease contraction, contracts the disease without drug 1 availability, contracts the disease with drug 1 availability but without buying drug 1, contracts the disease with drug 1 availability and buying drug 1, not buying drug 2, and buying drug 2. The parameter estimation in the previous section 5 illustrates the realization of the seven outcomes. We chose to illustrate outcome 7 which captures movement through the entire eight-period game in Fig. 1, while also illustrating why outcomes 1–6 do not arise with these parameter values.

As science, society, technology, and economic conditions change, the parameter values may change, which may cause any of the seven outcomes to emerge. For example, drugs may become cheaper or more efficient, viruses may mutate in unknown ways, pharmaceutical companies may become more efficient, donors may subsidize more or less, Nature may choose the probabilities differently, and persons may change balances they strike between risky and safe behavior. Insight into such changes may enable the players themselves, and non-modeled players such as healthcare workers, hospitals, governments, and international organizations to choose better strategies.

Various limitations exist for this study. First, as a useful first step we confine attention to N persons, one pharmaceutical company, one donor, and Nature, which means that we do not model other players in the health system, such as healthcare workers, hospitals, governments, regulators, politicians, and international organizations. Second, although the N persons differ in their utilities E_i_ of risky behavior, H_i_ of safe behavior, R_i_ when recovering from disease, and D_i_ of death, i = 1,…,N, we do not formalize the probability distributions for these utilities, which may change over time. Third, we consider one pharmaceutical company, which abstracts away competition between multiple pharmaceutical companies and market conditions which may impact prices, drug quality, etc. Fourth, we consider one parametric donor instead of multiple donors as strategic players potentially competing or facing a collective action problem. Fifth, we assume that Nature chooses disease contraction, recovery, mutation and death, some of which may be impacted by strategic choices made by non-modeled players.

Some of these limitations can be addressed in various ways in future research. First, research may classify different kinds of people according to age, sex, occupation, ethnicity, race, etc., and model probability distributions of various preferences and beliefs including risky versus safe behavior and how they recover from disease. Second, research may model more than one pharmaceutical company with different economic and scientific capabilities, preferences, and beliefs; may enable strategic choices by multiple donors, may incorporate more choices by Nature, and may model more players. Third, research may model different kinds of recovery from disease, depending on people’s different characteristics, economic capabilities, geographical location, etc. Fourth, research may model how disease contraction, recovery, and death impact and are impacted by drug production, drug quality, drug availability and costs, geographical distribution of drugs, and countries’ GDP, debt, productivity, income earnings, economic growth, societal conditions, and cultural preferences. Fifth, future research may model multiple donors as strategic players competing to donate in multifarious ways, may endogenize Nature’s parametric choices which may be extended beyond those considered in this article, e.g. by considering proliferation of virus mutation potentially spreading out of control. Sixth, the disease contraction probability with and without one or several drugs may be assumed to depend on multiple gradations and classifications of each person’s risky and safe behavior, the characteristics of persons, each person’s earlier medical history including exposure to infectious diseases, the number of persons having previously contracted the disease, and different kinds of matching of infected and non-infected persons. Seventh, various kinds of vaccinations prior to potential disease contraction may be incorporated into an analysis in combination with or matched against various kinds of drugs designed to ensure recovery after disease contraction. Eighth, the disease recovery probability may be endogenized by modeling the biological process by which viruses evolve [31, 34]. Ninth, research may model how people choose various kinds of behaviors, including potential vaccination if available, at different stages of drug development, i.e. at early stages where drugs may have uncertain quality and side effects, versus mature stages where the quality may have been improved and side effects ameliorated or removed. Tenth, research may incorporate different expectations by the players about future disease development, virus mutation, and drug availability. Eleventh, research may model how people may contract the disease at different points in time, and how the various players may make strategic choices at different points in time. Twelfth, research may tailor models to the specifics of various diseases. Thirteenth, future research should compile empirical data on a variety of infectious diseases including HIV/AIDS and Covid-19 to test the model in this article and future models that may be developed.

## Conclusion

An eight-period game is developed between N persons and a pharmaceutical company. Each person chooses either risky or safe behavior. Risky behavior may lead to disease, e.g. HIV/AIDS or Covid-19. The pharmaceutical company develops one or two drugs under various conditions. Drug 1 is developed if it is profitable, i.e. if sufficiently many persons contract the disease and choose to buy drug 1. Drug 2 is developed if the virus mutates, and if sufficiently many persons who have contracted the disease choose to buy drug 2. The donor chooses parametrically whether to subsidize the pharmaceutical company’s development of drug 1 and/or drug 2, and whether to subsidize each person’s purchases of drug 1 and/or drug 2. The donor’s choices thus impact both the pharmaceutical company’s choices and each person’s choices. This illustrates the game theoretic nature of the phenomenon. Nature chooses probabilistically whether a person with risky behavior contracts the disease, whether the person recovers without drug 1, whether the virus mutates, whether the person recovers with drug 1, whether the person recovers without drug 2, and whether the person recovers with drug 2.

Each person’s expected utility depends on seven outcomes, i.e. safe behavior, risky behavior without disease contraction, risky behavior with disease contraction and no drug 1 availability, drug 1 availability without buying drug 1, buying drug 1, virus mutation and not buying drug 2, and virus mutation and buying drug 2. The pharmaceutical company’s expected profit depends on three outcomes, i.e. no drug development (outcomes 1–3 for the persons), only drug 1 development (outcomes 4–5 for the persons), and virus mutation and drug 1 and drug 2 development (outcomes 6–7 for the persons). The donor’s expected utility also depends on these three outcomes.

Backward induction is applied to solve the game depending on conditions for the seven outcomes. The parameter values are estimated and exemplified in a procedure which applies HIV/AIDS data for prevalence, deaths, HIV expenditure, treatment costs, R&D costs and revenues, and HIV resource availability, for various countries. The model constitutes a tool or mechanism to comprehend a person’s choices between safe and risky behavior, whether to purchase one or several drugs, whether a pharmaceutical company should develop one or several drugs, whether a donor should subsidize drug development and drug purchases, accounting for how Nature chooses disease contraction, recovery, virus mutation, and death, with and without drugs.

## Data Availability

All data has been included in the article.

## Abbreviations

HIV: Human immunodeficiency virus infection
AIDS: Acquired immune deficiency syndrome
R&D: Research and development
ART: Antiretroviral therapy
UNAIDS: The Joint United Nations Programme on HIV/AIDS
HAART: Highly Active Antiretroviral Therapy (abacavir + didanosine + lopinavir/ritonavir)

## Footnotes

1 https://www.amfar.org/about-hiv-and-aids/facts-and-stats/statistics%2D%2Dworldwide/, retrieved February 20, 2021.
